# Can Self-Administered Rapid Antigen Tests (RATs) Help Rural India? An Evaluation of the CoviSelf Kit as a Response to the 2019–2022 COVID-19 Pandemic

**DOI:** 10.3390/diagnostics12030644

**Published:** 2022-03-06

**Authors:** Marika Vicziany, Jaideep Hardikar

**Affiliations:** 1 National Centre for South Asian Studies, Monash Asia Initiative, Monash University, Melbourne, VIC 3800, Australia; 2 Rural India Project, National Centre for South Asian Studies, Monash University, Melbourne, VIC 3800, Australia; jaideep.hardikar@gmail.com

**Keywords:** COVID-19, Indian pandemic, rapid antigen tests (RATs), CoviSelf, CoWIN, digital divide, rural India, Indian villagers, poverty, discourse analysis, qualitative medical/health research strategies

## Abstract

This paper evaluates India’s first officially approved self-administered rapid antigen test kit against COVID-19, a device called CoviSelf. The context is rural India. Rapid antigen tests (RATs) are currently popular in situations where vaccination rates are low, where sections of the community remain unvaccinated, where the COVID-19 pandemic continues to grow and where easy or timely access to RTPCR (reverse transcription-polymerase chain reaction) testing is not an option. Given that rural residents make up 66% of the Indian population, our evaluation focuses on the question of whether this self-administered RAT could help protect villagers and contain the Indian pandemic. CoviSelf has two components: the test and IT (information technology) parts. Using discourse analysis, a qualitative methodology, we evaluate the practicality of the kit on the basis of data in its instructional leaflet, reports about India’s ‘digital divide’ and our published research on the constraints of daily life in Indian villages. This paper does not provide a scientific assessment of the effectiveness of CoviSelf in detecting infection. As social scientists, our contribution sits within the field of qualitative studies of medical and health problems. Self-administered RATs are cheap, quick and reasonably reliable. Hence, point-of-care testing at the doorsteps of villagers has much potential, but realising the benefits of innovative, diagnostic medical technologies requires a realistic understanding of the conditions in Indian villages and designing devices that work in rural situations. This paper forms part of a larger project regarding the COVID-19 pandemic in rural India. A follow-up study based on fieldwork is planned for 2022–2023.

## 1. Introduction

As the Indian COVID-19 pandemic enters a new phase in 2022 with the rising dominance of the highly infectious Omicron variant, the use of self-administered RATs is increasing in India and other countries as a way of relieving the pressure on stressed clinical and hospital settings that administer and analyse reverse transcription polymerase chain reaction (hereafter RTPCR) tests [1]. As a third pandemic wave emerged in India, on 10 February 2022, the Indian Council of Medical Research (hereafter ICMR) announced that a positive result from a self-administered RAT was the equivalent of a positive result from an RTPCR procedure and that repeat testing using the latter was no longer necessary [2]. In this paper, we evaluate the first self-administered RAT to receive official approval by ICMR some nine months earlier, on 10 May 2021 [3]—CoviSelf. ICMR is the ‘apex body in India for the formulation, coordination and promotion of biomedical research’ [4] and sits within the Department of Health Research (Ministry of Health and Family Welfare), Government of India. As the first Indian made, self-administered RAT to receive official recognition, CoviSelf deserves our scrutiny. This diagnostic device for home use was developed by the private Indian company Mylab Discovery Solutions Pvt Ltd, based in Pune in the state of Maharashtra [5]. The tone of the company’s press release of 10 May 2021 reflects the excitement surrounding the development of this innovative testing kit during increasingly desperate times [3]:

With CoviSelf^TM^, Mylab aims to make testing reach *the doorstep of every Indian* to help them fight the second and any subsequent waves of [*sic*] pandemic. *Now, any citizen can test for Covid-19 themselves*, isolate and seek treatment quickly. Early detection can help save thousands of lives and significantly reduce [*sic*] burden on hospitals. The test can be purchased without a prescription from local pharmacies and online channel partners [italics added by authors of this article]

The claims about delivering a diagnostic tool for containing COVID-19 to ‘every Indian … any citizen’ are ambitious and admirable but need to be tested. Our analysis is not based on a scientific or laboratory assessment of the accuracy of this testing device. Few reliable studies of RAT evaluations have been published; perhaps the best-known exception is the UK’s Cochrane Report, published in March 2021 [6]. Until detailed and systematic quantitative data are published regarding Indian RAT devices, the official list of ICMR-approved RATs is our only source of information about the four criteria used to validate and approve the RATs distributed in India, including those used in home testing [7]. Moreover, official criteria are not an exact guide to how RATs function in medical, health and home settings. In mid-January 2022, ICMR published a list of eight self-test kits that it had approved, in addition to another list of five that it did not approve [7]. At the top of the approved list appears CoviSelf by Mylab.

As the first government sanctioned, self-administered RAT for COVID-19, our analysis of CoviSelf also provides a benchmark for evaluating the others that have become available in India since 10 May 2021. However, a scientific appraisal of the sensitivity and specificity of the RATs in India is not our aim in this paper [8]. Rather, our evaluation is based on the extent to which the CoviSelf kit is compatible with the practical needs and concerns of Indian villagers. We do this by considering the characteristics of CoviSelf in the context of the socioeconomic circumstances in Indian villages. Some 66% of Indians live in rural areas [9], so the applicability of CoviSelf has widespread implications for addressing the country’s pandemic while India waits for additional medical technologies to become available—for example, anti-COVID medication [10]. Yet, there is nothing inevitable about the arrival of such drugs or the trajectory that the pandemic could take. Given this, lessons can be learnt about the longer-term contribution that self-administered RATs could make to villagers’ wellbeing by analysing the practicality of the CoviSelf kit. Mylab’s aim for CoviSelf to reach ‘every Indian and any citizen’ is best tested against the socioeconomic parameters that define villages, where the majority of Indians live.

Our paper provides a qualitative evaluation of CoviSelf within a broad socioeconomic context. In 2008, Sir Michael Rawlins stressed the importance of recognising the value of qualitative research for health interventions. He questioned the hierarchy of evidence that gave greater importance to randomised clinical trials as the basis for health decisions. ‘Observational evidence’ or qualitative data, including ‘expert opinion’, formed an important component of therapeutic recommendations, he said [11]. Rawlins’ advice has been taken seriously and new health-medical literature has emerged in response [12,13], including an appreciation of the value of analysing documents of both a textual [14] and non-textual kind. This article belongs to this growing school of thought regarding the significance of qualitative research and its role in supporting better health and medical initiatives. Our analysis sits within the framework of discourse analysis, using textual sources to analyse CoviSelf. The words and language of these texts constitute our data. Our interest in the socioeconomic context of new diagnostic medical devices was prompted by one of our engineering colleagues, who asked: ‘what is the point of us inventing diagnostic tools unless we know how these fit into the daily life and needs of ordinary people?’ [15].

## 2. RATs in Clinical Settings in India

We are not aware of any published research assessing the self-administered CoviSelf kit, so we turn to what is known about RATs in clinical settings during India’s COVID-19 pandemic. ‘Clinical settings’ are those in which trained professionals administer the test in either an institutional or private environment, including clinics, hospitals, schools, offices, prisons, military facilities etc. This experience speaks to the value of making self-administered RATs accessible to all Indians.

By April 2021, 49% of all the tests for COVID-19 in India used RATs, ‘principally the SD Biosensor test (SD Biosensor Standard Q COVID 19 Antigen test)’ [16]. This was in response to the second wave of COVID-19 in the early months of 2021, a wave defined by the Delta strain and the shift of the Indian pandemic from towns to villages. By contrast, during the first wave of COVID-19 between December 2019 and October 2020 [17], only about a third of the infections occurred in rural India, but that increased in 2021 to over 50 percent according to the State Bank of India [18]. By mid-May 2021, 69% of India’s 748 districts (the lowest administrative level in the country) reported rates of infection above 10% [19]. The spread of COVID-19 generated interest in RATs as a way of saving the time taken for testing by RTPCR [20] and keeping track of the pandemic [21]. Although many extra RTPCR laboratories were set up throughout India during the first wave, the establishment and running costs of molecular biological testing facilities can be a limitation during times of a raging pandemic. In Bihar and Uttar Pradesh, states where rural healthcare facilities are especially limited, RATs played an even larger role by accounting for 87% and 59% of all the tests, respectively [16]. The cost of a RAT test is almost half or less than the cost of an RTPCR, an added incentive in the poorer states of northern India.

One review of RATs in India stated that they greatly ‘helped us … in detecting and diagnosing COVID-19 at its early stage and also by large scale screening of communities residing in hot-spot areas with high incidence of disease’ [22]. The Cochrane Report confirmed the value of all the above factors when using RATs for widespread diagnostic purposes [23]. Another study spoke of the benefits of RATs involving ‘triage and emergencies’ needing priority diagnoses [24]. RATs have long been used for diagnosing other illnesses, such as pharyngitis caused by Streptococcus bacteria [25] and Bancroftian filariasis [26]. Despite this, before the pandemic, there was a tendency amongst some Indian medical professionals to see RATs as an unreliable diagnostic tool [27]. That view has changed.

Compared with the ‘gold standard’ of RTPCRs, RATs are less accurate, although they are highly valuable during a rapidly escalating health crisis. One recent study based on 2168 outpatient samples reported a false negative rate from RATs of 4.3% in the first wave of the pandemic and 8.1% in the second wave [17]. It is for this reason that negative test results from RATs still need to be confirmed by RTCPR tests. However, in desperate situations where the status of a person with a possible COVID-19 infection needs to be quickly established, the small margin of inaccuracy in RATs is not a prohibitive factor to their use. Detailed reports about ICMR-approved RATs are only available to state governments, but we found one published study of the first RAT named in the ICMR list of 6 January 2022 [28]. Gupta et al. evaluated the Standard Q rapid antigen detection test (produced by SD Biosensor, Inc., Gurugram) and showed that the test accuracy was 95.4% and the ‘overall sensitivity and specificity of the test were 81.8 and 99.6 per cent’ [23]. The authors concluded that RATs with these kinds of properties could help alleviate the pressure on emergency departments in hospitals by allowing ‘patients showing positive results [to be]… immediately triaged’, thereby reducing the risks of COVID-19 infection spreading to other patients and staff in waiting rooms. ICMR then issued an advisory to Indian hospitals saying that the test should be ‘adopted in [their] diagnostic algorithms’. Unfortunately, the use of RATs in clinical settings is seriously constrained in rural India, where medical and health professionals are in short supply [29]. In that scenario, the question is whether a self-administered COVID-19 RAT might make the benefits of new diagnostic technologies more accessible to villagers. The need for rapid diagnostic assessments of COVID-19 infections in rural areas is evident from the situation that emerged from the start of pandemic in India.

### The Urgent Need for Self-Administered RATs in Villages

The negative effect of the pandemic has arrived on top of a long-term agrarian crisis in India. Agriculture has not been profitable for decades, and many farmers have been driven to suicide [30]. Poor, small, semi-literate farming families (including those who lease or share crop land, marginal farmers and labourers) have chosen to migrate to the cities for casual employment. When the Indian government declared an immediate national lockdown on 24 March 2020 due to the pandemic, millions of migrant workers found themselves walking home over hundreds of kilometres along India’s railway lines, or catching buses, trains or any other possible conveyance. Of the estimated 139 million migrant workers, perhaps a fifth formed a wave of reverse migration as they returned to their natal village homes in search of sustenance and emotional security. Plans to assist them during the pandemic were constrained because the central government had no idea of the scale of the problem due to a lack of statistics. What we do know is that about 6.3 million migrant workers were taken to state transport hubs closest to their villages by special trains between May and August 2020 [31]. There, they were left to walk to their homes, which were often hundreds of kilometres distant. Perhaps three to four times that number travelled home by other means. Back in their villages, they often returned to subsistence farming.

The official data regarding the mortality and morbidity rates of COVID-19 in India are no better than the data on migrant labour, and government estimates have been criticised for their lack of realism. Alternative estimates suggest that the total cumulative deaths in India have been between 2.5 and 7 times higher than the reported number of less than half a million [32,33,34,35,36]. Estimates of the total number of rural deaths *from* COVID-19 are significantly lower than the real figures because India’s percentage of medically certified causes of death (MCCD)—based on the signing of the ‘Medical Certificate of Cause of Death’ by a medical professional—is about 20% [37] of all the deaths at the national level. This low level reflects the fact that the ‘Medical Certificate of Cause of Death’ is typically signed in urban hospitals and clinics where the majority of Indian doctors are based. What people die of in rural areas is largely unknown unless special surveys are conducted. Thus, we can assume that the rural deaths from COVID-19 accounted for much more than the reported 55% of the total deaths between 2020 and 2022.

The impact of COVID-19′s devastation was revealed on 5 May 2021, showing that, in the preceding week, COVID-19 had become India’s leading cause of death, outstripping ischemic heart disease (the previous leading cause of mortality) by more than 2.5 times [38]. In summary, Indian villages remain in desperate need of medical attention during the ongoing COVID-19 pandemic. Given the inadequacies of rural health provision (see Section 4.3.2), self-administered RATs hold out the promise of giving villagers timely and useful information about how to protect their health. Our earlier study of the positive attitudes of villagers to point-of-care blood testing at their doorsteps suggests that what holds back the health of rural dwellers is not their opposition to innovative allopathic technologies but their poverty and lack of access to adequate healthcare (see Section 4.3.2 and Section 4.3.3).

## 3. Materials and Methods

This article employs qualitative data and methods to evaluate the self-administered Indian rapid antigen test CoviSelf.

### 3.1. Qualitative Framework

Content analysis and discourse analysis are two of many qualitative research methods that can be applied. The former assesses the frequency with which certain words, phrases or metaphors appear in a particular documentary text. This approach would not suit our purposes because we wish to understand the structure of the main document rather than focus on the frequency of particular words. Hence, we employ discourse analysis, where discourse is defined as ‘contextually sensitive written and spoken language produced as part of the interaction between speakers and hearers and writers and readers’ [39]. In our analysis, the speaker or writer is the producer of CoviSelf and the hearers or readers are the users of it.

### 3.2. Our Main Data Source

**Figure 1:**
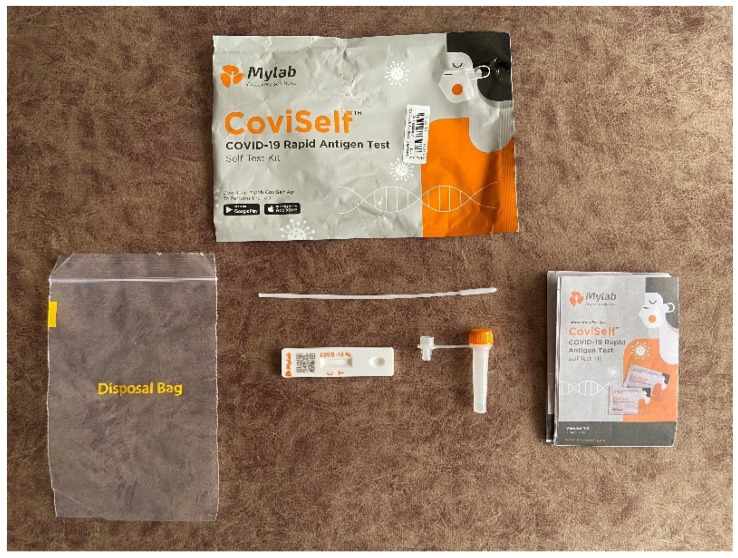
The components of the self-administered CoviSelf RAT kit. (Showing in clockwise order from the bottom left-hand side: the plastic bag for disposing of used items, the CoviSelf package, the instruction leaflet and, in the centre, the sterile nasal swab, the test card and the pre-filled extraction tube.) Source: Ms Anusha Kesarkar-Gavankar, photograph of 18 November 2021.

The main document we have analysed is the text of the printed ‘patient information leaflet’ that is part of the six components of the CoviSelf kit (Figure 1). Throughout this article, we refer to this document as the ‘instruction leaflet’ or the ‘leaflet’ [40]. Mylab calls this leaflet the ‘user manual’. We downloaded the English language version of the leaflet from the Mylab website [41]. We treat the leaflet as an agent reflecting the urgent diagnostic needs thrown up by the COVID-19 pandemic in India. As the first officially approved, self-administered, Indian made rapid antigen test for COVID-19, the text of this document has its own ‘potency as well as capacity’ [42]. We argue that the information embedded in the leaflet does not constitute ‘inert data’ but rather that the data are directed to particular social actors [42] who, in turn, interact with the data when they use the kit. That interaction involves the user of the kit reading the data (in the form of instructions) in the leaflet, interpreting it, interpreting the results of the COVID-19 test and interacting with any external agents who may be involved in the testing process—for example, any person helping the user to administer the test or the Government of India database that seeks to capture the user’s test results.

Our discourse analysis involved a close reading of the language of the instruction leaflet by checking for the three things required to effectively communicate the instructions to the consumer: first, we interrogated the text to see whether the assumptions it made about the user’s handling of the testing device were reasonable; second, we asked whether the language of the leaflet was clear, consistent and non-technical; and, third, we evaluated those sections of the leaflet that instruct the user about the IT components of the kit. From the start of our analysis, we were struck by the two main characteristics of the leaflet: it addresses both the test component and a special IT feature.

The leaflet was defined by us as the main document because it ends up in the hands of those who purchase the CoviSelf RAT kit. For this reason, we read the text multiple times to cross-check our findings, test out alternative interpretations of the data and resolve any inconsistencies that emerged. In reporting the ‘Results’ of our analysis below, we arranged our findings according to the sequence involved in using the self-administered CoviSelf RAT in order to place ourselves in the situation faced by a user. In evaluating the leaflet, we engaged in the ‘core activity’ of discourse analysis by thinking and categorising the ‘…actions, intentions, characters, events….’ [40] as revealed by this document.

### 3.3. Contextualising Our Main Data Source

An important part of our research method was to acknowledge that the meaning of the leaflet is not self-evident. The reader of the leaflet is not a passive, predictable sponge that merely absorbs the prefigured information provided in the printed text. Rather, particular readers construct the meaning of the instruction leaflet within specific cultural and socioeconomic contexts that vary from user to user. In other words, there is a process of negotiating taking place between the text and context, and it is this that enables the reader to construct meaning. Early in our evaluation, discourse analysis compelled us to ask where the CoviSelf diagnostic device sat within the broader framework of India’s IT revolution. For instance, which Indian consumers had smart phones capable of downloading the necessary app referred to in the leaflet? Mylab’s press release of 10 May 2021 stressed the benefits to Indian citizens of the kit’s features because they could now buy the diagnostic device ‘without a prescription from local pharmacies and online channel partners’, and, in using the kit, the consumer would promote the traceability of the pandemic through the company’s ‘AI-powered mobile App’ [3]. Our ‘Results’ below question the assumption that lies behind Mylab’s statement. We ask, does ‘every Indian and any citizen’ have access to the information technology that Mylab has taken for granted?

### 3.4. Our Second and Third Data Sources

These questions led us to the second data source—a group of documents produced by the Indian media, professional associations and the Supreme Court of India about India’s digital divide. The digital divide stressed the disadvantages suffered by rural residents in accessing electronic information.

Following the logic of discourse analysis, we then widened the context for understanding the CoviSelf leaflet even further by using a third data source, namely our own ‘expert opinion’, as documented in peer-reviewed, published research about living conditions in Indian villages. Fieldwork in central India before the pandemic showed us that villagers were receptive to point-of-care blood testing at their doorsteps [43]. We assumed, therefore, that there might be positive responses to the idea of using the self-administered CoviSelf RAT kit because the demand for modern medical interventions is driven by the desire to prolong longevity. This may well be a universal motivation, but our findings remind us that the specific cultural context, in Prior’s words, renders the ’unremarkable, remarkable’ [42]. With this third data source, we explored the interplay between the digital divide in rural India and the multiple constraints faced by villagers in accessing timely health and medical care.

### 3.5. The Limitations of This Study

With the ongoing COVID-19 pandemic, it has not been possible to undertake fieldwork because the desperate circumstances facing rural residents prevents them from participating in time-consuming, in-depth interviews. This is the main limitation of our present study. On the other hand, a discourse analysis of the three data sources (specified above) allowed us to refine our research questions and hypotheses for future research on village responses to the pandemic. In-depth interviews in rural areas during the next two years will constitute the fourth and final data source for an expanded study of the current paper. The locus of this extended study will build on our existing work in Wardha District (Maharashtra), the site of our research on point-of-care blood testing technologies. That earlier period of fieldwork was informed by our prior analysis of classical and modern Hindu texts that suggested that there were unlikely to be any cultural objections to modern diagnostic blood technologies. Our hypothesis for further fieldwork (in 2022–2023) on self-administered RATs in rural India questions the value of CoviSelf in villages. As a result of the present paper, we have come to the view that self-administered RATs for detecting COVID-19 infection would be more suitable to villagers if they did not include an IT component and if they were free. This hypothesis is not based on any questions about cultural resistance to the innovative CoviSelf device but rather doubts about its current practicality and cost. The urgent need for self-administered RATs in rural India suggests that investing in the redesign of this diagnostic device is well worthwhile.

## 4. Results

Our results are discussed under three main headings. Firstly, we report the findings from our discourse analysis of the main document, namely the kit’s printed instruction leaflet. In the second part, we explain the findings from our analysis of the data regarding India’s digital divide and its impact on rural India. In Section 3, we use our own data from previous studies of Indian villages. All three parts locate the CoviSelf kit within the wider socioeconomic context of Indian villages in conformity with discourse analysis, which seeks to understand how particular readers construct the meaning of different kinds of documents.

### 4.1. The CoviSelf Instruction Leaflet

Our analysis of the language of the leaflet focuses on four issues affecting the practicality of this self-administered RAT, but we begin our discourse analysis by considering the structure of that document. After that, the next two findings deal with the processes by which a test result is obtained, and the last two findings report on different aspects of the IT components of the kit. The data take the form of the leaflet’s instructions to the reader. Where appropriate, short comments are made about the practicality of the text in terms of its limitations and benefits.

#### 4.1.1. The Structure of the Instruction Leaflet

The layout of the leaflet is an important consideration in our discourse analysis because it tells us how the information provided to the user has been structured by Mylab, i.e., the order of priority given to various points. The leaflet takes the form of a folded, double-sided sheet of printed paper measuring 21 cm × 57 cm. One side is in English and the reverse side in Hindi. The leaflet is also available in eight other Indian languages representing different linguistic regions. The online version of each leaflet is identical in information and size to the copy included in the CoviSelf kit. The text is accompanied by diagrams illustrating all the instructions and how to read the positive, negative and invalid results. The only exceptions are the text in column one (both top and bottom panels, see below) and the bottom part of columns seven and eight, stating the FAQs (frequently asked questions), the limitations of the procedures, Mylab’s contact details and a QR code that can be scanned to access a video demonstration about how to use the kit.

Both sides of the leaflet are divided into eight columns, each with two panels (top and bottom). When folded, the leaflet measures about 10 cm × 7 cm. Column one, top panel, is taken up by the front cover of the leaflet, and the panel beneath it explains the kit’s intended use, kit storage and stability and the principles of the test; this last section explains some of the science behind the test device. Column two, top panel, is headed ‘Kit Contents’ and names the six components that make up the kit. The panel beneath that explains the ‘test preparation’, which includes washing and thoroughly drying the hands. The explanatory video that can be downloaded by some users says that a table top should be sanitised so that the contents of the kit can be laid out [44]. No such instruction appears in the leaflet.

Between the top and bottom panels is a central box that states: ‘Download the Mylab CoviSelf App from Play Store/App Store’. Column three, top panel, instructs the user to open the CoviSelf App and activate it; the bottom panel has instructions for preparing the pre-filled extraction tube. Column four is named ‘Step 1’ and instructs the user how to collect the nasal sample. Column five, designated ‘Step 2’, has instructions for placing the swab into the pre-filled extraction tube. ‘Step 3’ appears at the top of column six and tells the user how and where to place the sample on the test card. ‘Step 4’, beneath ‘Step 3’, asks the user to take a photo of the test card and ‘*wait for the App to analyse and display your Covid-19 test results*’ (authors’ italics). Under ‘Step 4’ appear instructions for disposing of the waste once the test has been completed.

Columns seven and eight, the leaflet’s last two columns, have two sections: the top explains the appearance of the test card when positive, negative or invalid test results appear. The bottom section provides additional information to the user: three FAQs are listed, five limitations to the procedure are stated and there is a QR code that can be scanned if the user wants to watch a video demonstration of how to use the kit. The bottom strip across both columns gives the name of the manufacturer and contact details.

Our analysis of the structure of the leaflet suggests that the order in which the instructions are arranged encourages the reader to use the IT component and to depend on it for ‘analysing’ the test results. Instructions about using the IT features are given at the start and end of the leaflet. The first instruction appears prominently in the middle of column two and asks the user to ‘Download the Mylab CoviSelf App’. This is followed by instructions in column three saying ‘*Open the Mylab CoviSelf App and fill in the credentials*’. After that, the user is told to scan the QR code on the test card. More significantly, ‘Step 4’ is presented to the user as the last step in the sequence of actions needed to complete the testing procedure. It asks the user to take a photograph of the test card and ‘*wait for the App to analyse and display your Covid-19 test results*’. However, the test card has already completed both of these functions: it has analysed the nasal sample thanks to the bioactive ingredients used in the test process and it has displayed the test result. In the instruction leaflet, there is a section that explains how the test card tells users whether they have a positive, negative or invalid test result. However, these explanations appear at the end of the leaflet, just before the FAQs section in columns seven and eight. A more logical order would have been for information about the test results to be labelled ‘Step 4’. The function of uploading a photograph of the test card to the app could then have been labelled as an optional ‘Step 5’. This new ’Step 5’ would also need to give the user an explanation for why the photograph of the test result should be uploaded to the app. In other words, the user should be given the choice of taking or not taking a photograph of the test result that has appeared on the test card. Instead, the instructions and current layout of the leaflet create the misleading impression that the IT functions of the kit are an integral part of the testing process. The true purposes of the IT functions are analysed below (Section 4.1.5). We also address the risks that these pose.

#### 4.1.2. The Ease of Using the Kit (the Relevant Headings in the Instruction Leaflet Are Steps 1, 2 and 3)

After scanning the QR code on the test card, the user is told to remove the pre-filled extraction tube from its packaging, hold it erect and tap the tube so that the ‘extraction buffer settles at the bottom of the tube’. The user is warned to be careful not to spill the contents of the pre-filled extraction tube—but no reason is given for this, so the user has no way of knowing the critical nature of this instruction. The fluid in the tube contains the reagent needed for testing the nasal sample. ‘Step 1’ then follows: it involves removing the swab from its packet by holding the tail end opposite the swab head. The swab head goes into the nostrils and so should not be touched because that could risk contamination. It is rolled five times in each nostril to collect cell samples.

Removing the swab from its package with one hand may be difficult if the user is already holding the pre-filled extraction tube in the other hand. Thus, there is a risk of spillage and contamination if the tube or swab are put down on a non-sterile surface. A second person could help by holding the pre-filled extraction tube, but, if that person has not washed and dried their hands thoroughly, there is another risk of contamination. In any case, instructions about this alternative approach are not given in the leaflet; it is assumed that the user is nimble.

‘Step 2’ comes next. On removing the swab from the nostrils, the swab head is inserted into the pre-filled extraction tube. The bottom of the tube needs to be pinched while the swab stick is swirled ten times to ensure that the nasal sample mixes properly with the pre-filled liquid. After that, the swab stick needs to be broken ‘at its breakpoint’, but this point is hard to see. The lower half of the swab, with the swab head, remains inside the tube. The top broken off part is placed into the disposable bag to be thrown away at the end of the testing process.

**Figure 2:**
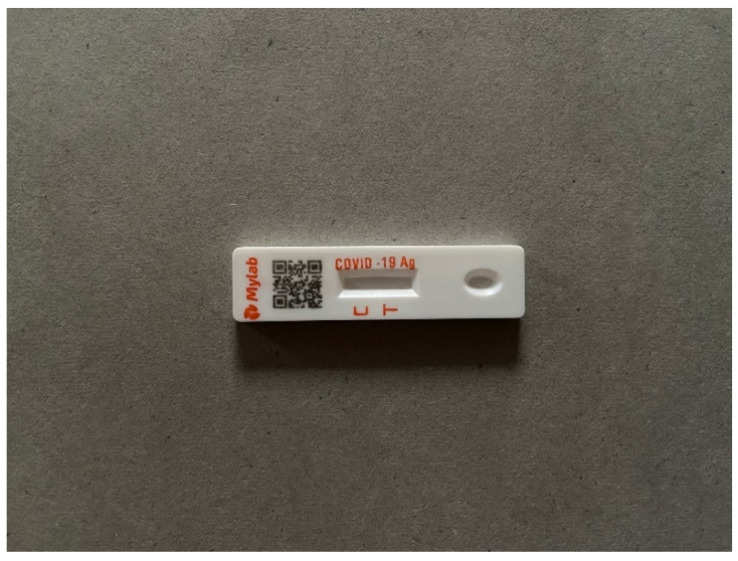
The CoviSelf test card. (From the far left: the QR code, the control point C, the test point T and the sample well for receiving the nasal mixture). Source: Ms Anusha Kesarkar-Gavankar, photograph of 18 November 2021.

Juggling these different tasks might not be so hard for an experienced user, but first-time users risk spilling the tube’s contents, contaminating the swab head or not collecting sufficient material from the nostrils. Nevertheless, supposing all has gone smoothly, the swab head is now inside the pre-filled extraction tube, which is then sealed with the attached cap. The test can now move to ‘Step 3’. The user is instructed to press the bottom part of the tube and place two full drops of the liquid into the sample well at the far end of the test card near the letter T (in Figure 2, the well is located on the far right of the image). Within 10 to 15 min, a test result should appear in the test window.

While waiting for the test results, the user can place all the discarded items into the disposable bag and ‘disinfect all surfaces that the specimen may have touched’ in addition to washing their hands after throwing the bag into household waste (bottom of column six). Soap and water are the best disinfectants against the coronavirus, and soap is readily available in Indian villages. However, what villagers might not know is that effective cleaning requires the user to scrub their hands ‘for at least 20 s’ [45].

#### 4.1.3. Reading the Test Result and the Clarity of the Leaflet’s Language (the Relevant Headings in the Instruction Leaflet Are Positive Result, Negative Result and Invalid)

The user can read the results on the test card, which has two letters in the centre beneath the test window: C and T. C is the ‘control’ point and T is the ‘detection’ point. If the test result is *positive*, two lines appear in the test window located in the centre of the card—one line next to the letter C and the second line next to T. The T line can be any shade of pink or purple, but all shades, no matter how light, indicate a positive test result. If only one line appears next to the letter C and there is no line next to the letter T, it means that the test result is *negative* for the virus. If no line appears in the test window next to the letter C, the result is invalid. The text should, however, clearly state that the test has failed.

The language in which the above information is expressed in the leaflet is complex and technical. For example, in the case of a positive test result, the leaflet reads: ‘If both the quality control line ‘C’ and the detection line appear, novel coronavirus antigen has been detected and the result is positive for antigen’. Antigen is not a well understood term in most countries, and neither is the notion of ‘detection line’ clear. However, the labels next to the diagrams illustrating the appearance of a positive and negative result are better: the ‘detection line’ is described as the ‘T test line’.

If a negative test result appears, the ‘symptomatic’ user is advised to immediately proceed with an RTPCR test because ‘RAT[s] are likely to miss [a] few positive cases presenting with a low viral load’. We have inserted the word ‘[a]’ into this quotation because, without it, the actual sentence says the opposite of what is intended. This problem in the English language version of the leaflet does not, however, occur in the Hindi version.

The leaflet also uses inconsistent terminology that can confuse users. For example, the test card is also called the test device or cassette, the pre-filled extraction tube is also called the pre-filled buffer tube and the liquid in the tube is called an extraction buffer and the antigen buffer.

#### 4.1.4. How to Respond to the Test Results (the Relevant Headings in the Instruction Leaflet Are Positive Result and Negative Result)

From the above account, it is obvious that the CoviSelf kit is a stand-alone device that has the capacity to tell the user whether they are or are not infected with the COVID-19 virus. However, the kit contains another component—an IT factor, which is problematic beyond our analysis of the misleading importance given to the app in the instruction leaflet (see Section 4.1.1).

The leaflet states that all positive results are ‘true positives’ and the user is advised to follow ‘home isolation and care as per the ICMR and Ministry of Health and Family Welfare protocol’. A website address with further instructions is provided for the user. All users lacking access to the internet will be excluded from further information—and we are speaking of millions of Indian citizens.

The advice is clearer in the case of a negative result. Here, the leaflet tells a user who has symptoms but receives a negative result to isolate at home and get an RTPCR test as soon as possible. Despite the misleading English language text explaining why more testing is required (see Section 4.1.3), users are familiar with the acronym RTPCR because, during the pandemic, such tests have become a routine procedure for hospital admissions. Nevertheless, users may not see the point of having an RTPCR test if their CoviSelf test results are negative. There is no compelling explanation in the leaflet to justify the expense and inconvenience of visiting a clinic or hospital to get an RTPCR test. The leaflet only states that a low viral load could mean that some positive cases are missed, but the words ‘low viral load’ are not automatically meaningful to most readers, who will not know about the science of virus testing.

More importantly, the user is not encouraged to take *another self-administered test* if the first result is negative. If the viral load is low at the time of the first test, a second test could pick up a true infection if the viral load has increased. There has now been considerable research and experience to show that, in the words of Richard Hatchett, ‘the antigen tests are less sensitive if you give just one. But if you can do it in a sequential way, they become cumulatively as sensitive as a PCR’ [46].

If the user is a villager, the advice to have a follow-up RTPCR test involves a time-consuming and expensive trip to an urban medical institution (see Section 4.3.2 below). ‘Isolating at home’ in the case of symptomatic individuals is also advice that is virtually impossible to follow as, in most villages in India, a whole family lives in a single room. In such cases, isolation at home actually increases the risk of all family members becoming infected. More fundamentally, individuals interpret the word ‘symptomatic’ very differently depending on their tolerance for risk. In India, recognizing the symptoms for COVID-19 is also difficult because the virus mimics the signs for many other illnesses—for example, other respiratory conditions accounted for a total of about 15% of Indian deaths between 2010 and 2013 [47] and much higher levels of morbidity.

#### 4.1.5. The Test Results and the Objectives of the ICMR (the Relevant Section in the Instruction Leaflet Is Step 4)

Instructions about the IT component of the kit are stated at the beginning and end of the leaflet. Prior to conducting the test, the user is asked to download the Mylab CoviSelf app from either Google Play or the App Store; a mobile phone message is then received asking the user to enter their personal details and scan the unique QR code printed on one end of the test card (on the left hand side of the image in Figure 2). The personal details are: name, age, gender, address, pin code, mobile and Indian ID number (or *Aadhar* card number). Except for the Indian ID number, which is an ‘optional’ detail, all the information is stated as being ‘mandatory as per ICMR guidelines’. Once the user has completed these tasks, they are ready to conduct the test. The result should appear within 15 min. The user is then asked to take a photograph of the test result and ‘*Wait for the App to analyse and display your COVID-19 test results*’.

There are two problems with these instructions. First, making the personal details ‘mandatory’ sounds ominous even if ICMR has no way of enforcing this. In Indian villages, such statements are taken seriously (see Section 5 ‘Discussion’ about ‘*sarkar*’). Secondly, it is misleading to state that the test result for COVID-19 infection is being analysed by the Mylab app. As already explained above, the result of the test appears within 15 min on the test card. What the leaflet should be saying is that, once the test result has been photographed, it is uploaded to Mylab records and then forwarded to the ICMR database, which forms part of the records of the Government of India. Equally important, there is no provision in the leaflet for the user to give their consent to sharing their personal details or test results with the government.

The role of ICMR is only explained on the Mylab CoviSelf website, where there is a section headed FAQs. From this, we can learn about the destination of the uploaded test result in response to the following question: ‘What are the benefits of reporting the result to ICMR?’ The following answer is given [48]:

ICMR is the regulatory body in India which is responsible for the curb of COVID-19 pandemic along with other important regulatory bodies. Reporting the results helps the body and authorities curb the spread of the disease. It thus becomes our moral obligation to help ICMR by reporting our test result data.

It is significant that the above FAQ and answer do not appear in the user leaflet, although the FAQ section in that leaflet takes up a total of 20 lines, suggesting that there was enough space to include the five lines about the role of the ICMR database. Helping the government to collect better records of COVID-19 infection is a worthy objective, but it could end up being counterproductive to do so without the prior agreement of the user (see Section 5 ‘Discussion’). Moreover, it is just as important to protect the right of Indian citizens to keep their personal details private and their medical records confidential [49].

From the user’s perspective, is there any benefit to activating the IT component of CoviSelf? The analytical methods of discourse analysis tell us that the answer to this question depends on who the user is.

After the user sends a photograph of the test result to the Mylab CoviSelf app (and ultimately to the ICMR) via their mobile phone, they receive a notification of their COVID-19 status. This can be beneficial for several reasons. First, it might reassure those users who are uncertain about their reading of the results on their test card. The response from the Mylab app gives them a statement about their test result, and this validation enables them to act accordingly. Second, many middle-class Indians have used this statement as evidence of their COVID-19 negative status when boarding domestic flights and entering hotels. Such certification has enabled some professionals and holiday seekers to be mobile inside India [50]. One hopes that such travellers have been cautious and only travelled when they were asymptomatic. However, as already noted, everyone’s tolerance for, and interpretation of, ‘symptoms’ differs.

These benefits are counterbalanced by the multiple problems that arise from trying to use the IT components of CoviSelf. Many urban users have complained about the difficulties (see Section 6 ‘Conclusions’). It is regrettable that the instruction leaflet conflates the two components of CoviSelf, i.e., the test and the IT functions, because this gives users the impression that the kit will not work without activating the IT features. In rural India, the inadequate provision of healthcare services suggests that the potential value of a self-administered COVID-19 RAT is high, but the IT component compromises the device’s usefulness for the reasons we explain in the next two sections.

### 4.2. India’s Digital Divide

India’s digital divide is part of a global problem and influences the value of the CoviSelf kit because it contains an IT feature. In 2002, the Secretary General of the UN spoke of the digital divide as the widening gap between the ‘haves’ and ‘have nots’ in accessing the new information and communications technologies and the dangers this posed by excluding the world’s poor from the ‘emerging knowledge-based global economy’ [51]. The key technologies for such access were the internet and mobile phones. Two years earlier, UN members agreed that one of the Millennial Goals was to ‘ensure that the benefits of new technologies, especially information and communication technologies, are available to all’. The data we have used to provide the general context for understanding the implications of this digital divide for CoviSelf come from the following sources: the Indian press, a 2021 report by the Internet and Mobile Indian Association (hereafter IAMAI) and a court case by the Software Freedom Law Centre before the Supreme Court of India in 2021.

#### 4.2.1. The Indian Press

The phrase ‘Indian press reports about India’s digital divide’ revealed over 46 million hits on the Google search engine. A report in 2019 from the *Economic Times* stated that India had an estimated 450 million smart phone users and about 550 million users of ‘feature phones’ [52]. A feature phone is defined by Kantar IMRB/MMA as ‘A mobile phone that incorporates a fixed set of functions beyond voice calling and text messaging such as limited web browsing and e-mail, ability to play music *but generally cannot download apps from an online market place*’ [authors’ emphasis] [53]. Whilst most mobile feature phones allow users to access the internet for entertainment, they typically lack the capacity to support complex internet apps, such as the one used by CoviSelf. This places the innovative CoviSelf device beyond the majority of Indians despite the hopes that Mylab has of reaching all citizens.

#### 4.2.2. The Internet and Mobile Association of India (IAMAI) Report of 2021

A more fundamental problem is the unreliability of telecommunication signals in many parts of India, especially rural areas [54]. The 2021 IAMAI report on internet usage in urban and rural India shows unsurprisingly that states with relatively good telecommunications infrastructure have a higher percentage of active internet users (hereafter AIUs) amongst their population (e.g., 61% in Maharashtra) than states lacking such facilities (e.g., 24% in Bihar) (Table 1). This reflects the level of urbanisation: 45% of Maharashtra’s population live in towns and cities compared with 11% in Bihar [55]. For the whole of India, AIUs represent about 31% of the rural population compared with 67% percent of urban residents (Table 1). The percentage of people who have what is often called ‘connectivity’ in India may be less than this as many AIUs might have more than one phone and more than one internet account.

#### 4.2.3. Civil Society and the Supreme Court of India

The exclusion of the rural poor from internet access is well known within Indian elite circles, yet, despite this, the Government of India created the CoWIN app in early 2021 to make it easier for Indian citizens to get appointments for vaccinations against COVID-19. The idea behind this innovative app was to stop citizens standing in long queues for vaccinations—thus saving time, reducing the risks of cross-infection and taking the pressure off clinics, hospitals and vaccination centres. Unfortunately, when the new system of appointments was announced on 18 April 2021, it also became mandatory for all Indians aged from 18 to 44 to make online appointments via the CoWIN app. The Software Freedom Law Centre (hereafter SFLC) in New Delhi wrote to the Indian Ministry of Health objecting to the mandate and, after being ignored, raised a Sou Moto case in the Supreme Court of India against the ministry [56]. The SFLC argued that the CoWIN app [57] failed to address the ‘digital exclusion’ of Indians who lacked appropriate mobile phones and internet access and it also failed to protect the privacy of citizens [58]. Justice Chandrachud (Supreme Court) described the government’s assumptions behind the CoWIN portal as ‘far-fetched’ and ‘exclusionary of the rural areas’ [59].

The objections against the CoWIN app (before the mandatory requirements were removed) apply equally to the IT component of CoviSelf. However, there has been no public outcry against CoviSelf because buying the device in India is a matter of personal choice rather than something mandated by the state. Nevertheless, this should not blind us to the fact that the digital divide deprives millions of citizens from the benefits of this self-administered RAT. The government’s failure to address the needs of villagers in the provision of self-administered RATs is inconsistent with its strong campaign to improve rural hygiene since 2014 through the Swachh Bharat Mission to end, for example, open defecation and the risk of faecal infection [47]. This contradiction in the government’s rural development programs cannot be addressed in this paper because the topic is too large and complex, but, in the following section, we consider the practicality of CoviSelf for Indian villagers.

### 4.3. The Conditions of Life in Rural India

In extending the context in which to understand the benefits and limitations of CoviSelf, we have drawn on our own research published in peer-reviewed international journals and books. Vicziany has published on the poverty and marginalisation of *Dalits* (former ‘untouchables’) [60], the family planning program [61,62], food security [63,64,65], Koli villages in Mumbai [66] and rural health [29,43,47], including work on the potential of point-of-care blood testing in villages [29,43]. Hardikar has reported extensively on questions of rural poverty, co-authored work with Vicziany on poor farmers, agriculture and health [29,43] and has published two books on India’s farming crisis [30,67].

#### 4.3.1. Village Poverty and Employment

The bulk of our research has dealt with poor, semi-literate people in marginalised and socio-economically disadvantaged households. Our most recent research in Wardha District (Maharashtra) involved 36 in-depth interviews with farming groups defined by various criteria: a high proportion of families living below the poverty line, households with very little irrigated land, poor farmers, landless villagers and tribal or *Dalit* families [43]. Our findings showed that poor rural households are preoccupied by three major concerns. Their first priority is to secure a source of income through any kind of employment- even if they farm; supplementary sources of income are important, in particular through short- or long-term migration to urban centres. Those who remain in villages seek to diversify their income locally—for example, by serving as government agents for the public food distribution system, working as linesmen for electricity companies or providing local taxi services.

Our understanding of the drivers of poverty in rural India and the constraints it imposes on the capacity of villagers to avail themselves of the benefits of modern technologies was confirmed by the Supreme Court’s judgement in the CoWIN case (see Section 4.2.3). The judges noted that the minimum internet tariff plan would equal ‘4–5% of the month’s income’ of urban and rural people living below the poverty line [68]. Accessing internet data is, therefore, something that they cannot afford given that the poor are also heavily indebted. The burden of poverty extends into all aspects of life, including the lack of consumer power to buy the CoviSelf kit that retails for about Rs. 250. Given that the daily wage for a farm labourer at the top end of the range of agricultural jobs (i.e., ploughing and tilling) is about Rs. 365 [69], the cost of CoviSelf is equal to 68% of the daily wage that sustains a whole family. For the unemployed and those living below the poverty line of Rs 1316 and Rs 896 *a month* in urban and rural India, respectively [68], the expense of the CoviSelf kit is unthinkable. Moreover, the benefits of all self-administered RATs are greater if used more than once over a number of days to check the onset and end of infection. Such costs are beyond the means of India’s poor whether they are marginal farmers, labourers, the under-employed or the unemployed.

Our research on rural poverty is supported by other quantitative data showing that 27.9% of India’s population is suffering from multidimensional poverty [70], defined as multiple and overlapping conditions of deprivation. The MPI (multidimensional poverty index) goes beyond previous attempts to measure poverty using simple headcounts and monetary estimates of the poverty line. As a result, the MPI replaced the Human Poverty Index (HPI) of the United Nations Development Programme in 2010. The data show that interstate variations in poverty in India are stark and support our previous reference to the large regional disparities between, for example, Maharashtra and Bihar. The proportion of the population in these two states that is multidimensionally poor is 15% and 52%, respectively [71]. Poverty weighs especially heavily on disadvantaged social groups throughout India (such as the *Dalits* and tribal people), with five out of six Indians belonging to these communities suffering from multidimensional poverty [72]. Rural economic insecurity is the overall context in which we need to evaluate the practicality of CoviSelf, the affordability of mobile phones and internet access and the future of self-administered RATs for villagers.

#### 4.3.2. Village Health and Diagnostic Medical Interventions

The second priority of Indian villagers is their health. India’s medical system is a plural one, ranging from trained allopaths (i.e., doctors trained in the ‘western’ medical system) and Ayurdevic-Unani practitioners to herbalists, soothsayers, spirit men, midwives, gurus, spiritual mediums and ‘quacks’. The Maharashtra Medical Association, for example, has been engaged in a campaign to marginalise all but allopaths, yet the plurality remains because there are insufficient allopaths willing to serve in rural areas. Even in states that have seemingly adequate rural health infrastructure (such in Maharashtra), the human resource constraints are serious. The result is that villagers bypass government rural medical institutions and travel considerable distances to clinics and hospitals in or near towns [43]. While this is costly, rural residents have the satisfaction of getting a more timely and reliable diagnosis of their condition, a prescribed treatment regime and admission to hospital in urgent cases.

The healthcare sector is now dominated by private services, with many hospitals being part of larger systems of education involving private medical colleges. Providing medical training and degrees has become a lucrative business in India and attracted many entrepreneurs. The appearance of the private health sector has resulted in villagers experiencing a medical revolution, with entrepreneurs working to make their hospitals more accessible and affordable to the poor by offering ‘membership cards’ and using their political links to help residents in need of attention. In return, these entrepreneurs and politicians are rewarded when their beneficiaries vote for them in elections [43].

Yet, despite this, the shortage of medical and health professionals in rural areas has not been alleviated. The private entrepreneurs that have developed western based medical institutions are urban based. In the government’s rural health infrastructure there are large human resource shortfalls and vacancies relative to requirements [73]. At the village level in India, health care is left to what can best be described as a pre-modern private sector. One rare study reported that the village level private sector accounted for 68% of local healthcare and that 75% of villages had at least one health care provider. However, of the 3373 providers surveyed, only 8% were allopaths with an MBBS qualification and 24% were AYUSH providers with degrees in alternative medicine, namely in Ayurveda, Yoga, Unani, Siddhi and Homeopathy. The remaining 68% lacked any formal training [74]. In other words, the size of the private sector reflects the poor relationship that exists between villagers and government funded institutions. It does not mean that the private sector provides better health and medical services, even if these are more accessible.

For all the above reasons, villagers with life-threatening health problems go straight to the nearest hospitals, even though these are expensive. ‘Free’ treatment in government institutions, subsidised health care in the private sector and the support of an emerging insurance system have failed to reduce the out-of-pocket (hereafter OoP) expenses for inpatients and outpatients. Our study of the potential for point-of-care blood testing at the village level showed that there are many potential rural innovators because OoP costs are too high. Of the 36 villagers we interviewed between 2017 and 2018, 61% were receptive to the idea of introducing simple blood tests into their villages [43]—using bioactive paper, such tests would require only a few drops of blood and allow villagers to avoid the many costs incurred by travelling to town-based institutions. More generally, we discovered that medical innovations do not come as surprises to Indian villagers; their receptivity to blood tests at their doorsteps reflected their experience with modern medical practices, ranging from complex procedures, such as liver transplants and transfusions for accidents through to regular blood testing and treatment for genetic blood diseases [43].

Rural residents place a high value on their health, and, because of this, they are willing to spend large amounts of money on medical diagnoses and treatments to address their illnesses and increase their longevity. Many rural households go into debt to cover medical expenses. There is no evidence in the available literature or our fieldwork to suggest hesitancy arising from their distrust of modern medicine; rather, they may be distrustful of the government institutions charged with the delivery of rural healthcare.

#### 4.3.3. Village Religion and Modern Medicine

The third preoccupation of villagers is to ensure that their family and local religious traditions are respected, including the worship of goddesses. The COVID-19 pandemic has seen the proliferation of Corona goddesses. This could easily be misunderstood by non-Indian observers thinking that here is a contemporary example of Indian fatalism and attitudes opposed to allopathic medicine. However, our research showed that goddess worship, including blood sacrifices to appease village and household deities, sat comfortably alongside the demand for modern, scientific health interventions. One of the villagers that we met explained that her husband had died of alcoholism despite the fact that a goat had been sacrificed to the family deity on his birth. We asked her why the mother goddess, Firasti-Aai, had not provided her husband with lifelong protection against ill fortune. She exonerated the deity and blamed her husband:

He had good health but he brought it upon himself by drinking too much. How is our deity responsible for that? [43].

Powerful religious beliefs, in other words, do not cancel out personal responsibility. We found no evidence of any clash between local religious traditions and innovative medical practices.

The prudent course of action for believers wishing to prolong their lives is to propitiate the goddesses and simultaneously address their ill-health by resorting to appropriate medical solutions. Since independence in 1947, Indian governments have also promoted widespread immunisation for infectious diseases, and this has contributed to a decline in death rates. However, despite great demand in rural India, the present pandemic has been characterised by shortages of vaccines, oxygen, hospital beds, masks and drugs, such as remdesivir. There has not been any cultural resistance to these measures. For most of 2020 and 2021, vaccinations against COVID-19 in rural India lagged seriously behind those in urban areas, demonstrating the ‘urban bias’ that Indian development policies suffer from. By mid-May 2021, only about 15% of rural residents had received at least one dose of vaccine [75], partly because urban–rural disparities cause India’s cold chain to be unevenly distributed [76]. By early 2022, however, the vaccination gap had closed thanks to a new focus on villages, although urban–rural differences remain, with 79% of eligible urban residents fully vaccinated compared with 69% of rural dwellers in late January 2022 [77].

## 5. Discussion

The unique status of the India-made, self-administered CoviSelf RAT kit is based on official approval by ICMR. In the context of a pandemic, that places a special responsibility on the government to ensure that this diagnostic device can benefit all Indian citizens. The IT component of the kit fails this basic test because millions of people in India do not have access to the internet and thus the app embedded in the CoviSelf kit is out of reach for them. India’s digital divide has prevented universal internet access because there are too many poor households that lack the financial resources to buy sufficiently sophisticated mobile phones or pay for online information. There is also the matter of the privacy of the user for, as explained above, personal details are being entered into a Government of India database without prior consent from the individual users of CoviSelf. These realities fly in the face of Mylab’s objective of bringing CoviSelf to the ‘doorstep of every Indian … any citizen’, as stated in the company’s press release of 10 May 2021 [3].

Might the official approval of CoviSelf be defended by the argument that the privacy of the poorest Indian citizens cannot be compromised if they are unable to download the Mylab app or upload their test results?

Such a defence makes no sense for a number of reasons. First, villagers come and go between villages and towns, and they might well purchase a CoviSelf kit thinking that it will help to protect their families from infection. The poor are attracted to innovations that hold out the promise of a better life, and unsuitable decisions can be made as a result. Second, even if they cannot read the CoviSelf leaflet, villagers can go to a local ‘fixer’, who, for a small fee, will read the leaflet and try to handle the IT component of CoviSelf on their smart phone. This ‘solution’ has been reported for rural residents accessing other government apps [54]. Should this happen, the villager becomes caught up in all the limitations of the kit. When the fixer follows the leaflet, the first thing they will read is the instructions for downloading the Mylab app and the last thing they will read will be about sending the image on the test card to Mylab. The fixer might not realise that the test result is before them on the test card and that nothing further needs to be done to find out whether the user has tested positive or negative for the virus.

The self-administered CoviSelf RAT kit, therefore, fails two tests of importance in the world’s largest democracy: the right of all Indians to access new technologies approved by the Indian government and the right to privacy. The IT component of CoviSelf carries an additional risk, namely that it could create suspicion about the real purpose of this self-test diagnostic device and also about other government-approved medical technologies. If the government hopes to contain the spread of COVID-19 and harness self-administered RATs to that end, it should consider using local hospitals with good reputations in rural areas. For example, in Wardha District, we discovered that the Kasturba and Datta Meghe Hospitals are highly regarded by villagers for their excellent services, including rural outreach work [43]. It would not be difficult to involve the professionals from such institutions in the promotion and use of CoviSelf. Such professional backing could also help to disseminate more accurate information about the nature of COVID-19 and how to avoid infection. Trusted health professionals could explain to villagers the health risks posed by *symptomless* individuals who are nevertheless carrying COVID-19. Normally, villagers do not seek diagnosis or treatment for symptomless family members, as we know from the failures of the government’s anti-TB program [78].

The prerequisites for such outreach are twofold: make the kit free to all citizens and remove the IT component. Given the multidimensional nature and extent of Indian poverty, the first point is significant. The second point also matters if CoviSelf is to be trusted by villagers. The present design of CoviSelf is likely to raise the suspicions of villagers: if medical professionals are seen entering a villager’s personal details into a smart phone, it would soon become widely known that the test result and private information were being sent by Mylab to the Government of India. The Hindi word for government is ‘*sarkar*’, and, amongst India’s poor, it inspires fear rather than confidence. For complex historical reasons, ‘*sarkar*’ has never been a trusted institution in India. The fear would be not only about the possible uses of such data but also anxiety regarding some kind of government or police action in the event that they test positive.

## 6. Conclusions

Our evaluation of India’s first officially approved self-administered COVID-19 RAT kit uses the framework of discourse analysis to ask whether CoviSelf is of any practical value in rural India. Drawing on data from a wide range of qualitative, textual sources, we have identified a number of significant problems with the language of the instruction leaflet and the assumptions it makes about the living conditions in rural India; specifically, its promotion of the IT features of this diagnostic device betrays a lack of insight into why India’s ‘have-nots’ have been excluded from the information technology revolution.

The key lesson emerging from our evaluation is that, if CoviSelf is to be made more practical and accessible to Indian villagers, the IT functions should be removed and the kit made cost-free to users. Such changes would also automatically protect the privacy of the user and their test result. The ambitions of Mylab could well be realised by such adjustments, namely the production of a self-administered, Indian-made COVID-19 diagnostic device for every Indian. In removing the embedded IT features of CoviSelf, the instruction leaflet would need to be rewritten, and that would provide an opportunity to address the other language problems that we found in the text.

Such changes would also help frustrated urban middle-class users whose struggles with the kit are recorded on the Google Store website. Many users wrote that the kit was a waste of money, some suggesting that it was a scam, others that the instructions were wrong and that the test card was not working properly. One frustrated customer (Ramana G) wrote on 1 November 2021 [79]:

I had to take three tests and the result is still inconclusive, what is the problem. The 1st test …I couldn’t squiz [*sic*] the liquid onto sample level and I broke the well bottom and the lines so I exited the App. But then I tried the second time and did everything right and found that results were inconclusive as I scanned later than 20 min… I tried third time and exactly waited for 15mins and it’s the same problem.

Some three months later, another customer (Arjita Mukherjee) on the same website commented:

I followed all instructions yet I didn’t receive my results. Just said Invalid Casette, low server problem. Second, I understand that there might be server issue, then what’s the point of keeping another 15 min time interval when the whole kit gets invalid after 20 min. Just keep the test result appearance that’s it. Really disappointed. The manual rtpcrs are far safe and better I suppose.

This kind of feedback allows us to predict a villager’s likely experience with CoviSelf in its present form. The online customer reviews confirm our evaluation of the instructional leaflet—namely that there are serious communication problems with the language in the leaflet, the instructions to users and the IT features.

The potential for self-administered RATs in Indian villages, especially in the face of the highly infectious Omicron strain, remains undiminished. The majority of Indians live in rural areas where access to timely medical diagnosis, assistance and drugs is seriously constrained. Modifying the CoviSelf kit to remove the limitations that we have identified in this paper could assist with its equitable distribution as an effective, risk-minimising device against galloping infections in villages. Richard Hatchett has given a prescient warning at the height of the Omicron wave: ‘unpredictably, the virus appears to have the capacity to become, essentially, a pandemic at any time… access to diagnostics—and updated diagnostics, are absolutely critical to managing an infectious disease crisis’ [46]. Given this, the distribution of self-administered RATs such as CoviSelf should accompany vaccines and anti-viral drugs as an essential part of India’s pandemic planning.

## Figures and Tables

**Table 1:** Internet usage in urban and rural India in 2020 (based on active internet users (AIUs)).

Variables	All India	Urban	Rural
Population in millions	1433 mil	485 mil	948 mil
Active internet users (AIUs)	622	323	299
% growth in AIUs during last 12 months		4	13
% of AIUs in urban/rural India		67	31
Top 9 cities’ share of urban AIUs as a %		33	
Share of AIUs in villages with populations over 1000			85
Highest usage state in India: Maharashtra with highest % of AIUs relative to state population	61		
Lowest usage state in India: Bihar with lowest % of AIUs relative to state population	24		
Ratio of male: female AIUs		57:43	58:42
% AIUs using mobiles		100	100
% AIUs using PCs		22	13
% AIUs using other, e.g., tablets, smart TVs etc.		7	5
Average duration of AIUs on internet in mins		115	99
% of AIUs using internet for entertainment		96	96
% of AIUs using internet for Communication		92	87
% of AIUs using internet for social media		84	79
% of AIUs using internet for net commerce		59	30
% of AIUs using internet for online Shopping		43	13
% AIUs texting & emailing		87	79
% of AIUs voice & video messaging		54	57

Source: Collated from Kantar, *Internet Adoption in India: ICUBE 2020*, Internet and Mobile Association of India/Kantar: Delhi, India, June 2021, (https://images.assettype.com/afaqs/2021-06/b9a3220f-ae2f-43db-a0b4-36a372b243c4/KANTAR_ICUBE_2020_Report_C1.pdf, accessed on 20 February 2022).
